# Use of Web Conferencing Technology for Conducting Online Focus Groups Among Young People With Lived Experience of Suicidal Thoughts: Mixed Methods Research

**DOI:** 10.2196/14191

**Published:** 2019-10-04

**Authors:** Jin Han, Michelle Torok, Nyree Gale, Quincy JJ Wong, Aliza Werner-Seidler, Sarah E Hetrick, Helen Christensen

**Affiliations:** 1 Black Dog Institute University of New South Wales Sydney Australia; 2 School of Social Sciences and Psychology Western Sydney University Sydney Australia; 3 Department of Psychological Medicine University of Auckland Auckland Australia

**Keywords:** online focus group, young people, suicide, qualitative methods

## Abstract

**Background:**

There is an increasing interest in engaging people with lived experience in suicide prevention research. However, young people with suicidal thoughts have been described as a “hard-to-include” population due to time, distance, stigma, and social barriers.

**Objective:**

This study aims to investigate whether conducting synchronous Web conferencing technology–based online focus groups (W-OFGs) is a feasible method to engage young people with lived experience of suicidal thoughts in suicide prevention research.

**Methods:**

Young people aged between 16 and 25 years and living in Sydney, Australia, were recruited through flyers, emails, and social media advertisements. The W-OFGs were established using a Web conferencing technology called GoToMeeting. Participants’ response rate, attendance, and feedback of the W-OFGs were analyzed to determine whether the W-OFG system is feasible for suicide prevention research. Researchers’ reflections about how to effectively implement the W-OFGs were also reported as part of the results.

**Results:**

In the pre–W-OFG survey, 39 (97.5%) young people (n=40) chose to attend the online focus group. Among the 22 participants who responded to the W-OFG invitations, 15 confirmed that they would attend the W-OFGs, of which 11 participants attended the W-OFGs. Feedback collected from the participants in the W-OFG and the post–W-OFG survey suggested that online focus groups are acceptable to young people in suicide prevention research. Considerations for selecting the Web conferencing platform, conducting the mock W-OFGs, implementing the risk management procedure, inviting participants to the W-OFGs, and hosting and moderating the W-OFGs as well as a few potential ethical and pragmatic challenges in using this method are discussed in this study.

**Conclusions:**

The Web conferencing technology provides a feasible replacement for conventional methods, particularly for qualitative research involving vulnerable populations and stigmatized topics including suicide prevention. Our results indicate that this modality is an optimal alternative to engage young people in the focus group discussion. Future studies should compare the data collected from the Web conferencing technology and conventional face-to-face methods in suicide prevention research to determine if these two methods are equivalent in data quality from a quantitative approach.

## Introduction

### Background

There is an increasing consensus regarding the importance of involving people with lived experience of suicidal thoughts in the design of health care services and interventions [1,2]. However, concerns about ensuring the safety of those with lived experience as well as structural barriers to attending interviews or focus groups lead to substantial challenges for researchers when using conventional face-to-face methods to engage this population [3]. The conventional face-to-face focus group, a well-known means for collecting qualitative data in health care research [4], has long been criticized for its limitations with regard to the number of the topics that can be included in a given time as well as restrictions imposed by the geographical location where the group is conducted [5]. Lack of freedom in choosing the participatory locations can be a barrier to participation, particularly for people with lived experience of suicidal thoughts. They may worry about insufficient protection of their anonymity due to the chance of being coming from the same community [6]. These reasons could be especially true for young people, who frequently report privacy concerns and lack of money to travel long distances as two major barriers to participating in mental health research and services [7-9].

### Online Focus Groups

The rapid development of digital technology has given rise to a group of new methods for collecting qualitative data in health care, including online focus groups that can be delivered either asynchronously (ie, participants contribute to the conversation at different time) through messenger services or forums [10-12] or synchronously (ie, participants contribute to the conversation at the same time) through chat rooms or Web conferencing technology [13,14]. Through these technologies, online focus groups can operate like conventional face-to-face focus groups by providing real-time communication for multiple users from different geographical locations while saving costs for both transcription and travel [15]. Online focus groups also have greater potential than conventional face-to-face focus groups for participants to maintain anonymity. User identities can remain hidden [16] as long as a unique identification code is assigned and quoted as part of their response. This potentially increases the willingness of participants to exchange opinions about sensitive or potentially taboo topics, and there is evidence to support this in respect to sexuality [14] and domestic violence [17]. Furthermore, online focus groups may provide more “equal” chances of participation, as potentially stigmatizing personal details (eg, social status and educational background) are not as readily available. Such sociodemographic factors have been found to potentially impede equal participation in face-to-face interviews, as some participants with a perceived lower status may defer from those who were perceived to have a higher social standing [18].

Online focus groups may be particularly appealing to young people, who are familiar and comfortable with communicating and engaging in the virtual world and online apps [19,20]. Burton and Bruening [18] suggest that gathering the opinions of young people through a medium that they are familiar and comfortable with “can potentially produce data that sheds more light on the experiences of these participants than survey research or even traditional focus groups do.”

### Web Conferencing Technology–Based Online Focus Group

One type of synchronous online focus groups, the Web conferencing technology–based online focus group (W-OFG), provides real-time communication between participants and moderators across various geographical locations similar to an Internet chat room. Moreover, the W-OFG has advantages over the internet chat room in enabling immediate and spontaneous communication through real-time videos and sounds via various devices that can be used, such as full-motion Webcam, microphones, and speakers. Compared to the internet chat room, the W-OFG mimics the operating environment of the conventional face-to-face focus group better by capturing more nonverbal and paraverbal cues (ie, tone, pitch, and pacing of voices), which has been suggested to be vital for focus groups [15].

In the last 4 years, the W-OFG has been used to engage male victims of partner abuse [17], university students [21], and geographically dispersed health professionals [22-25] in research. In these studies, delivering focus groups via the W-OFG was well received by both participants and researchers due to its convenience and anonymity. Importantly, the W-OFG has a similar level of data richness [26] and group interactivity [21] as conventional face-to-face focus groups.

Although several limitations of the W-OFG have been noted in the literature, including underrepresentation of the overall community due to computer usage and availability [27] and a potential high no-show rate (ie, over 50% of the people signing up but not attending the W-OFG) [22,28], the W-OFG serves as a viable alternative to comparable conventional methods. However, there have not been any published W-OFG studies in the suicide research literature, and therefore, the feasibility or efficacy of this methodology in this area is not clear.

This research study is conducted as the first step in a participatory approach to the design and development of a smartphone app for managing suicidal thoughts of young people. This paper focuses on describing young people’s preferences of focus group settings, acceptability of the W-OFG, and researchers’ methodological reflections on the procedure of the W-OFG. The content of the data acquired using the W-OFG and the online surveys will be the subject of a separate paper. The findings reported in this study are expected to provide the first empirical implications for the feasibility of using online interviewing methods to involve vulnerable young people in suicide research practice.

### Aims

Given the lack of published studies on W-OFG in suicide prevention research, this study aims to (1) examine young people’s preference of focus group settings (face-to-face or online) in suicide prevention research; (2) describe the W-OFG procedure in a reflective way; (3) determine whether the W-OFG is feasible for suicide prevention research by analyzing young people’s response rate, attendance, and feedback; and (4) discuss the ethical implications with regard to privacy and safety of the W-OFG in suicide prevention research.

## Methods

### Participant Eligibility

Participants were first screened online for their eligibility before being invited to an online survey and to attend focus groups. Participants were deemed eligible if they (1) were aged between 16 and 25 years; (2) were located in Australia; (3) were fluent in English; (4) were able to attend face-to-face focus groups in Randwick or Sydney’s central business district, or were willing to do online focus groups on the scheduled dates; and (5) had lived experience of suicidal ideation. Participants were excluded if they (1) had been diagnosed with schizophrenia or a related psychotic disorder, (2) had experienced suicidal thoughts in the past 2 weeks, or (3) had attempted suicide in the past month.

### Participant Recruitment

**Figure 1:**
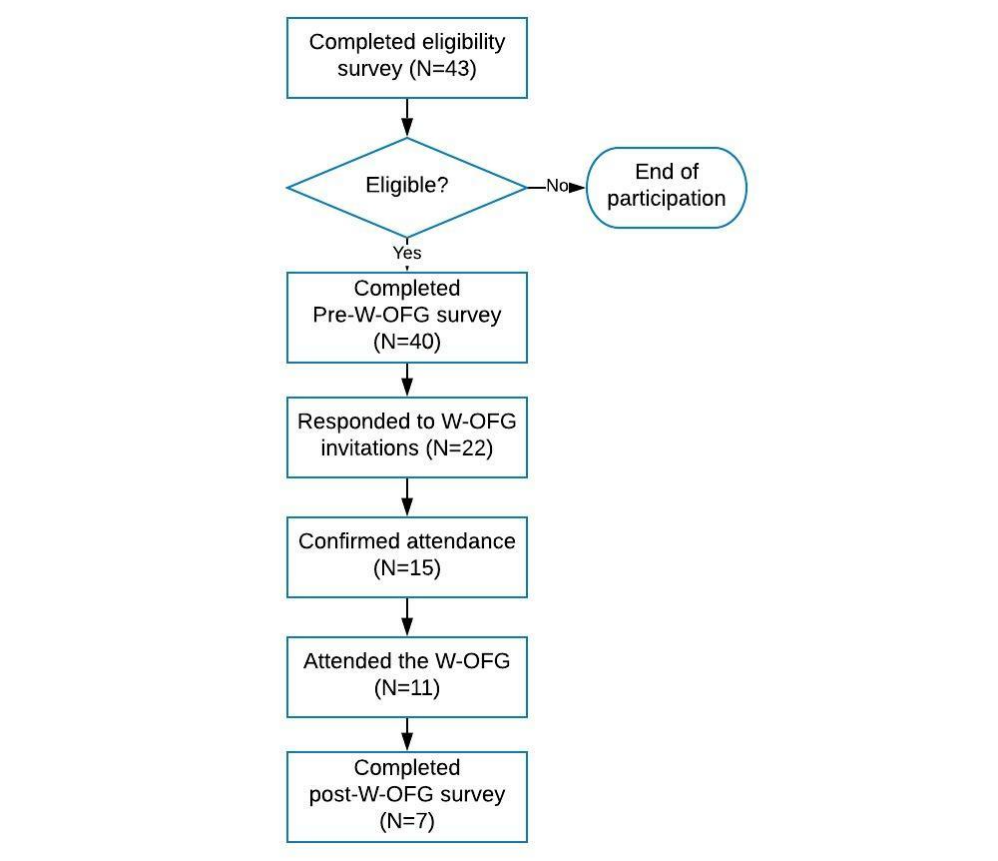
Diagram of participant flow. W-OFG: Web conferencing technology–based online focus groups.

Participants were recruited through online advertisements on Facebook and the Black Dog Institute (BDI; a medical research institute) social media channels, including Facebook, Twitter, and Instagram, through the registered volunteer network at the BDI and through flyers at the Black Dog Clinic and Headspace at Bondi Junction and Camperdown in NSW, Australia. Multiple methods were used to maximize recruitment. Targeted Facebook advertisements were used to recruit young people aged between 16 and 25 years in Sydney who were interested in mental health issues suggested by following one of the following social media accounts, including Beyondblue, Headspace, Lifeline, RUOK Day, and SANE Australia. The advertisement included a brief introduction of the study design, the eligibility criteria, and mental health support information such as a contact number of Lifeline Australia. Content was slightly adjusted to meet the word limit of different channels (ie, Facebook, Twitter, and Instagram). Thirty (75.0%) participants reported hearing about the study from Facebook advertisements; 11 (27.5%) from the BDI social media channels; 6 (15%) from the BDI clinics and Headspace; and 4 (10%) from Instagram. An AUD $100 electronic gift card was given to the participants who completed the focus group in acknowledgment of the time and internet expense related to participation. Figure 1 illustrates the participant flow in this study. Details about the procedure were described under the relevant section of the results.

### Study Design

The research study used mixed methods to analyze the feasibility of the W-OFG in suicide prevention research. Data were drawn from two online surveys (before and after the W-OFG), and one relevant question was asked at the end of the W-OFG.

The pre–W-OFG online survey was designed to collect participants’ characteristics and preference for the type(s) of focus groups. Participants were asked to choose the focus groups they were prepared to participate in, including conventional face-to-face focus groups at two local sites in Sydney, and the W-OFG, through a select-all-that-apply question. Participants were notified that the W-OFG was only available for young people aged between 18 and 25 years (ie, not for individuals aged 16-17 years) according to the ethics requirements. Other questions included those on participants’ age; sex; nationality; living and relationship status; education; severity of their suicidal thoughts assessed by the Suicidal Ideation Attributes Scale [29]; the number of lifetime suicide attempts; history of suicidal thinking; general help-seeking intentions measured by the General Help Seeking Questionnaire [30]; and smartphone use habits including the devices, installed apps, and average time spent using apps.

The W-OFG focused on young people’s use habits of smartphone apps, their preference of app features and designs, and how they may use apps to manage suicidal thinking. At the end of the W-OFG, participants were asked about their experience with the process by responding to a question, “We will take this chance to ask how you’ve found this focus group method. Any comments on that?” All participants’ responses to the question are listed in the results.

The post–W-OFG online survey was designed to ensure the quality of the W-OFG and was optional. Questions about the areas of improvement of the W-OFG, motivations of participation, and undisclosed comments about the ideas raised in the focus group were asked in the post-W-OFG survey. The areas of improvement of the W-OFG were summarized and reported in the results. The results also included researchers’ reflections about the procedure of the W-OFG.

### Ethics Statement

The study received ethics approval from the Human Research Ethics Committee at the University of New South Wales (protocol number: HC180646). The research was conducted in accordance with the ethical principles of the Declaration of Helsinki.

## Results

### Participant Characteristics

The online eligibility survey was automatically closed after attaining 40 eligible participants. The total sample (n=40) had a mean age of 21.2 years (SD 2.4 years, range: 16-25 years). The majority (n=37, 92.5%) were female, and all were Australian and spoke English at home as their primary language. No participants identified as Aboriginal or Torres Strait Islander. Half (n=20, 50.0%) were not married or in a relationship, and close to half (n=19, 47.5%) lived with their parents or family. Similar values have been found among those who responded and confirmed that they would attend the W-OFG (Table 1). For the participants who attended the W-OFG (n=11), the average age was 21.3 (SD 1.9) years; all of them were female, 7 (63.6%) were not married or in a relationship, and 3 (27.3%) lived with their parents or family. The target number of participants were recruited within 1 day of using the online recruiting methods.

**Table 1:** Characteristics of the participants grouped by participation.

Characteristics	Completed the online survey (n=40)	Responded to attend the W-OFG^a^ (n=22)	Confirmed the attendance (n=15)	Attended the W-OFG (n=11)
Age, mean (SD)	21.2 (2.4)	21.8 (2.0)	21.5 (2.1)	21.3 (1.9)
Female, n (%)	37 (92.5)	20 (90.9)	14 (93.3)	11 (100)
Never married and not in a relationship, n (%)	20 (50)	10 (45.5)	8 (53.3)	7 (63.6)
Living with parents or family, n (%)	19 (47.5)	7 (31.8)	5 (33.3)	3 (27.3)

^a^W-OFG: Web conferencing technology–based Online Focus Groups.

### Pre–Web Conferencing Technology–Based Online Focus Group Survey: Examining Participants’ Preference for the Types of Focus Groups

A total of 39 (97.5%) young people chose to attend an online focus group. Only one individual (2.5%) chose the conventional face-to-face focus group as the preferred format. As the majority of the surveyed young people preferred the online focus group, it was implemented in this study. One participant aged below 18 years was not eligible for the W-OFG. The moderator (NG) contacted the participant and discussed the opportunity to have a one-to-one interview to participate in the study. The participant expressed an understanding but declined the opportunity.

### Selection of the Web Conferencing Platform

In this study, a Web conferencing technology called GoToMeeting [31] was used to run two focus groups with young people who had lived experience of suicidal thoughts. It is one of the two online meeting software platforms provided by the University of New South Wales. The other option was Zoom Video Communications. The researchers tested the two Web conferencing platforms according to the modified eight criteria from Tuttas [22] for the online meeting software, which were as follows: (1) ability to accommodate up to 10 participants in the group, (2) ability to run a focus group for up to 1.5 hours, (3) ability to support real-time audio and full-motion video imaging, (4) ability to support audio and video recording of the focus group, (5) ability to restrict the access to recordings within the research team, (6) no more than moderate technical competency required from participants, (7) no obligation to purchase and install the software, and (8) provision of access for only invited parties to enter the meeting space. Both GoToMeeting and Zoom fulfilled all criteria with the exception that Zoom only allowed 40 minutes of the meeting to be recorded for free. Therefore, GoToMeeting was selected to host the focus groups in this study.

### The Mock Web Conferencing Technology–Based Online Focus Group

As suggested by Irani [27], a mock W-OFG was conducted by the researchers in a private conference room with adequate lighting. The W-OFG moderators became familiar with the functionality of both the participant and the moderator screens under the assistance of an internal information technology helpdesk supporter. The questions for the focus group were reviewed and practiced in the mock W-OFG to estimate how long the focus group might take to complete. The accessibility of the W-OFG was tested using multiple devices, including smartphones and laptops. Automatically generated audio records and transcripts were reviewed for completeness and accuracy. An action plan was developed to manage any potential technical difficulties that may arise, and a risk management procedure was outlined to manage any psychological distress experienced by participants.

The mock W-OFG yielded important insights that required attention. First, a number of technical issues were identified. These included a slide presenting major themes that organized the questions to be asked in the online focus group and background music, indicating that the participant had successfully logged in to the system. Dialing into the focus group using mobile phones needed to be prohibited, as participants would not have been able to see the screens shared by the moderators: Only Web-based devices such as smartphones, desktops, and laptops could be used to access the W-OFG. The provision of quick guidance about the major features of the conferencing system from the moderator was helpful regardless of participants’ Web literacy level. In addition, participants needed to be advised in advance that they could use the Webcam if they wanted to. Participants could text the moderator in the chatlog anytime during the focus group to inform the moderator that they were experiencing psychological distress or when they wanted to give further comments in response to questions. The information was only accessible by the moderator. The lessons we learned from the mock W-OFG were used to inform the implementation of the W-OFG.

### Risk Management Procedure

The risk management procedure was adapted from a protocol of a study among male suicide survivors who discussed their positive strategies to prevent and manage suicide in face-to-face interviews and focus groups [32,33]. It is composed of general risk management, identification of at-risk participants, and management of at-risk participants.

First, general risk management included reminding all participants at the beginning of the W-OFG that a clinical psychologist was available in the instance of distress. They could message the moderator anytime if they felt distressed or planned to withdraw the W-OFG. Second, participants were reminded to be mindful of the spoken content and limit their answers to how technologies or other skills had helped them manage prior suicidal experiences. Third, participants were asked to be respectful of each other’s opinions and were informed that all the content shared in the W-OFG was confidential and sharing the Webcam content was optional. Following this, time was allocated to participants to read the participant information sheet and raise any questions or concerns orally or by message. Participants needed to provide oral consent or written consent by message before the W-OFG. Finally, at the end of the W-OFG, the moderator checked how participants were feeling, and a question regarding self-care activities was asked to all the participants.

At-risk participants were identified based on statements made by the participant during the course of the focus group, which indicated distress, or by direct disclosure of distress to the moderators. Visible signs of distress were not available during the W-OFG, as participants preferred not to use their Webcams, which is a limitation of this approach. If participants became distressed during the W-OFG, the moderator would check if they needed a break or support from the clinical psychologist. If the participant required a break, one of the moderators would keep the discussion going, while the other moderator would stay in touch with the participant. The moderator would ask the participants if they could contact support persons for the participants. At the end of the W-OFG, the at-risk participants would be messaged individually to check their safety. If they felt calm, they would be notified that they could contact the research team later if they needed help. The research team would check in on them the next day if this was consented to. If the participant still felt distressed, the moderator would offer to contact their carers or health professionals. They would be followed up the next day if they agreed. All incidents related to identifying “at-risk” participants would be recorded in the risk-management log.

### Invitation to the Web Conferencing Technology–Based Online Focus Group

Eligible participants who provided written consent were contacted by the researchers to arrange an online focus group time using the contact method they chose in the online survey. Thirty-eight (95%) of the young people chose to be contacted by email, while only two (5%) chose to be contacted by phone. No participants wanted to be contacted by short message service. Initially, three timeslots were provided to the participants. Twenty-two (55%) participants responded to the W-OFG invitations. Each timeslot received 7-10 expressions of interest (EOI). The participant flow is presented in Figure 1.

The two timeslots that received the most EOIs were confirmed with the participants by email. At this stage, 15 participants confirmed that they would attend the W-OFGs, by email or phone. All other participants who indicated that they could not attend either of the two focus groups received an email notifying them that they had been put on a waiting list. After the two W-OFGs took place, JH and NG reviewed the audio records and agreed on content saturation. A thank-you letter was sent to the participants on the waiting list after the completion decision was made by the research group.

A unique identification code was allocated to each participant to replace their real names when logging in to GoToMeeting. The email also contained brief instructions about how to use GoToMeeting and a digital copy of the participant information sheet in which a detailed description of the study was provided. A group of help resources (eg, contact number of Lifeline) was provided at the bottom of the email. The researcher’s contact information, research project Webpage link, and ethical approval number were also included in the email. It was also emphasized in the email that participants could withdraw their consent and discontinue participation at any time without any consequences.

Two reminders of the focus group were sent to the participants by email 1 day and 1 hour before the W-OFG. Of the participants who confirmed that they would attend the W-OFG, approximately 70% (5/7 and 6/8) attended each of the two focus groups. A similar level of attendance was noted in the face-to-face focus groups [34]. One of the four participants (25%) who signed up for the W-OFG but did not show up reported a time conflict. The other participants did not respond to the emails. All communication between the participants and the research team was monitored by the researchers with training in clinical psychology or counselling. Following the Recovery Oriented Language Guide [35], words such as “excluded” or “ineligible” were intentionally avoided in the communication, and the emails were worded using person-centered language. Specifically, all young people who did not meet the selection criteria received a personalized end-of-study message, including the reasons why they were ineligible at that moment. Participants who were put on the waiting list or did not attend the W-OFG were followed up by emails to notify the progress of the study and check their safety.

### Hosting and Moderating the Web Conferencing Technology–Based Online Focus Group

The two focus groups were led by the same moderators (JH and NG). Upon initiating the focus groups, the moderators framed the discussion with a message around respect for different opinions and equal opportunity of participation. This helped foster a safe environment and facilitated a connection between the moderator and participants in the absence of visual cues. Subsequently, the participants were given enough time to go through the digital participant information sheet and raise questions if they wanted to before they were asked to provide individual verbal consent for participation. The use of the Webcam was optional, and no participants chose to use the Webcam functionality.

Participants were asked to mute their microphones during the focus group to improve recording quality and avoid the interruptions in the conversation. After each question, participants who wanted to express their ideas were required to unmute their microphone and wait until their number was called by the moderator. The times each participant was invited to speak were balanced by the moderator. Participants could also text the moderator in the chatlog during the W-OFG if they were not invited to respond to the question or if they preferred input in this way. Two participants chose to use the chatlog function due to privacy concerns and a technical problem with the microphone.

Finally, the W-OFG concluded with a question focused on self-care with the aim of leaving participants feeling empowered and on a positive note. The rationale for this originated from Sharkey et al [36] who, in their online research among vulnerable young people, highlight why participants need to consider studies carefully. Therefore, the final question posed by the moderator was whether the self-care activity participants were going to engage in activities upon completion of the focus group.

### Acceptability of the Web Conferencing Technology–Based Online Focus Group

At the end of the W-OFG, young people were asked about their experience with the process by responding to a question, “We will take this chance to ask how you’ve found this focus group method. Any comments on that?” Three participants responded to this question orally:

I think it’s been great, like it hasn’t been glitchy at all the whole time.

I was quite surprised at how easy this was to use, so I think it definitely a good way, especially to stay anonymous I think.

I [think the process is] way better than Skype.

The feedback provided by participants at the end of the W-OFG was in accordance with the comments received from the post W-OFG survey. Seven of the eleven participants filled in the survey and responded to an open-ended question: “Can we do anything to improve the way we run future focus groups?” No particular area of improvement was indicated by the participants. The exception to this was that one participant suggested that it might be better to provide the questions before the W-OFG.

### Records of the Web Conferencing Technology–Based Online Focus Group

GoToMeeting automatically generated the records of audio, the chatlog, and the automated transcript after the W-OFG. The original audio records of the W-OFG were transcribed through a paid transcribing service. The quality of the audio record was acceptable. On an average, 12 per 10,000 words were labelled as inaudible. The record of the chatlog was of good quality without missing information. User identification and time were precisely recorded. However, compared to the transcription from the paid transcribing service, less than 50% of the content was obtained by the automated transcript provided by GoToMeeting, indicating that the transcription from the former should be preferred over the latter.

## Discussion

### Principal Findings

This study reports on the process of using Web-based conferencing systems to host the W-OFG among young people with suicidal thoughts. It provides the first report on using this method in suicide prevention research and provides a basis for further development of conducting synchronous online focus groups with people experiencing sensitive or stigmatized mental health issues such as suicidal thoughts.

Although previous studies have suggested that the “no-show” rate is often high (over 50%) in the W-OFG [18,22,28], around 70% of the participants who agreed to participate attended the scheduled W-OFG. This number is similar to the level of attendance reported in virtual focus groups (81% by video and 69% by chat) in a previous study, but lower than that in face-to-face groups (94%) [37]. Provision of incentive, delivery of reminders, themes of the focus group, and the target population may have influenced the attendance rate, but warrant further investigation. The whole process was described as surprisingly easy by the participants. Although the W-OFG is an emerging method with great potential to include participants in a less costly way, there are some issues that need to be acknowledged and addressed before implementing the W-OFG in people with suicidal thoughts.

First, we need to acknowledge that participants who participate in the W-OFG are likely to be an unrepresentative sample of the community, as the W-OFG can only include people who have access to the internet. The technology itself can be a barrier. For example, the W-OFG may be a less favored means of participating in research by elderly individuals who are unfamiliar with digital technologies or by people living in areas where the internet is expensive or less accessible. However, because of these criteria, the W-OFG may also be particularly advantageous for research topics related to digital technologies, such as developing a smartphone app, as these topics require participants to convey experiences of using technology.

Second, the safety of participants is paramount in the context of the W-OFG. The absence of nonverbal cues and lack of environmental information might hamper the immediate assessment of respondent behaviors and emotions during the focus group. This increases the difficulty of providing support during the W-OFG. For example, the meaning of periods of silence was difficult to decipher, that is, it was difficult to know if it was a sign of disengagement or distress, or simply a result of participants taking some time to think about their response to the question. Therefore, it was difficult to determine whether it was necessary to remind participants of the option to write privately to the moderator or that a clinical psychologist was available in the instance of distress. As there is limited evidence about how to manage risk among vulnerable populations in online focus groups, we propose five considerations on the basis of our experience and Irani’s [27] work: (1) those conducting the online focus group consider the health status and the technological literacy of potential participants during recruitment; (2) those conducting the online focus group ensure participants know that they do not have to answer anything too painful or distressing or things that they do not want to share; (3) those conducting the online focus group create safety management procedures involving clinicians, allowing participants to withdraw at any time during the study and providing follow-up check-in if participants allowed; (4) those conducting the online focus group frame the discussion with messaging around respect for different opinions and equal opportunity of participation upon initiating the W-OFG; and (5) those conducting the online focus group conclude with the focus group by asking questions regarding self-care activities.

Third, although a potential strength of the W-OFG is to overcome potential barriers with regard to the physical location and the lack of privacy of face-to-face focus groups, there is evidence suggesting that participants in a W-OFG are less likely to elaborate on others’ opinions compared to participants in a face-to-face focus group [15]. We observed a similar tendency in our W-OFGs, as participants often used simple statements such as “I agree,” which can be simply substituted for nods in the conventional face-to-face focus group. In addition, it remains unknown if acknowledging others’ comments is due to peer pressure, which could lead to group polarization. On the other hand, it could represent the opposite with previous studies, showing that focus groups can be a positive experience for participants due to group membership and cohesiveness [38]. Therefore, further qualitative studies are needed to distinguish between these possibilities.

Finally, although no participant expressed any concern about privacy in the W-OFG, digital data collection via a video conferencing system could raise issues of privacy and data breach. Similar to any other type of information and communication technology, video conferencing systems have inherent vulnerabilities including issues such as intentional attacks from hackers or accidental security breaches due to user ignorance or misunderstanding. For example, most video conferencing systems allow automatic audio-saving online and locally. It is therefore the researchers’ responsibility to check the Web conferencing security documents provided by the video conferencing companies before using the system to minimize the chances of data being retrieved without permission.

### Limitations

Several limitations need to be addressed in this study. First, the Web conferencing platforms were compared and decided by the researchers. This could be improved by taking users’ preference into consideration. Furthermore, validated measures such as the System Usability Scale [39] can be used in the future to quantify users’ feedback of the system. Second, the majority of the participants involved in the study were female, suggesting that other strategies may be required to engage men. However, there is a broad issue of women being more likely to engage in research [40-42]. Overrepresentation of women in this study may also reflect the natural gender bias in suicidal risk (ie, females are more likely to ideate and attempt suicide) [43]. This sample was not representative but fulfilled the goal of attracting potential end-users to provide their perspectives on the app features that can help them manage their suicidal thoughts. Since the focus groups were also restricted to a small group of participants that had Web-based devices and could access the internet, the generalizability of these findings to the broader population of young people with suicidal ideation should be approached with caution. Lastly, no comparisons between the conventional face-to-face focus group and the W-OFG were made in this study. It remains unclear if these two types of focus groups generate similar quality data in suicide prevention research. Future studies may address this limitation by using the two methods in the same study and comparing the findings.

### Conclusions

Conduct of focus groups using digital technology is gaining popularity among researchers due to flexibility and functionality that traditional methods cannot logistically offer, including reaching out to those from diverse population groups and remote areas. Recent studies suggest that actively involving individuals with mental illness in the research process is beneficial to the participants [44]. This study provides preliminary evidence that the use of the Web conferencing technology can be a feasible replacement for conventional methods, particularly for research involving vulnerable populations and stigmatized topics including suicide prevention. Future studies need to compare the data collected from the Web conferencing technology and conventional face-to-face methods in mental health and suicide research to determine if these two methods are equivalent in data quality from a quantitative approach and to target respondents from disadvantaged social and demographic backgrounds to confirm the feasibility.

## Abbreviations

BDI: Black Dog Institute
W-OFG: Web conferencing technology–based online focus group
